# A new material to prevent urethral damage after implantation of artificial devices: an experimental study

**DOI:** 10.1590/S1677-5538.IBJU.2016.0271

**Published:** 2017

**Authors:** Salvador Vilar Correia Lima, Marcilio Romero Machado, Flávia Cristina Morone Pinto, Mariana Montenegro de Melo Lira, Amanda Vasconcelos de Albuquerque, Eugênio Soares Lustosa, Jaiurte Gomes Martins da Silva, Olávio Campos

**Affiliations:** 1 Núcleo de Cirurgia Experimental, Programa de Pós-Graduação em Cirurgia do Departamento de Cirurgia do Centro de Ciências da Saúde da Universidade Federal de Pernambuco, UFPE, Brasil;; 2 Serviço de Urologia do Hospital das Clínicas, Departamento de Cirurgia do Centro de Ciências da Saúde da Universidade Federal de Pernambuco, UFPE, Brasil;; 3 Departamento de Patologia, Centro de Ciências da Saúde da Universidade Federal de Pernambuco, UFPE, Brasil;; 4 Departamento de Cirurgia do Centro de Ciências da Saúde da Universidade Federal de Pernambuco, UFPE, Brasil;; 5 Departamento de Biologia Aplicada à Saúde, Laboratório de Imunopatologia Keizo Asami (LIKA), Universidade Federal de Pernambuco, UFPE, Brasil

**Keywords:** Urinary Incontinence, Urethra, ethyl-2-hydroxyethylcellulose [Supplementary Concept], Polysaccharides, Bacterial, Biocompatible Materials

## Abstract

**Objective:**

To validate the application of the bacterial cellulose (BC) membrane as a protecting barrier to the urethra.

**Materials and Methods:**

Forty female Wistar rats (four groups of 10): Group 1 (sham), the urethra was dissected as in previous groups and nothing applied around; Group 2, received a 0.7cm strip of the BC applied around the urethra just below the bladder neck; Group 3, received a silicon strip with the same dimensions as in group 2; Group 4, had a combination of 2 and 3 groups being the silicon strip applied over the cellulosic material. Half of the animals in each group were killed at 4 and 8 months. Bladder and urethra were fixed in formalin for histological analysis.

**Results:**

Inflammatory infiltrates were more intense at 4 months at lymphonodes (80% Grade 2), statistically different in the group 2 compared with groups 1 (p=0.0044) and 3 (p=0.0154). At 8 months, all samples were classified as grade 1 indicating a less intense inflammatory reaction in all groups. In group 2, at 8 months, there was a reduction in epithelial thickness (30±1μm) when com-pared to groups 1 (p=0.0001) and 3 (p<0.0001). Angiogenesis was present in groups 2 and 4 and absent in group 3. In BC implant, at 4 and 8 months, it was significant when comparing groups 4 with 1 (p=0.0159).

**Conclusion:**

BC membrane was well integrated to the urethral wall promoting tissue remodeling and strengthening based on morphometric and histological results and may be a future option to prevent urethral damage.

## INTRODUCTION

Urinary incontinence (UI) represents a urological problem of growing prevalence, affecting about 2.5 to 40% of individuals, and in addition to determining a high economic cost to the health institutions, reveals itself as a devastating phenomenon, implying physical and psychological misfortunes that affect the quality of life and self-esteem of the patient, contributing to social isolation from family, friends and sexual partner (1-3).

This is a symptom of various causes, and may be linked to bladder dysfunction (hyperactivity), weakness of the urethral rabdoesfincter (post prostatectomy radical injury or female UI) (4). The weakness of the external urinary sphincter usually comes from surgical trauma, neurological conditions and congenital anomalies (5).

Alternative treatments, conservative measures are provided (water restriction, use of absorbent, penile clamp, exercise and pelvic floor biofeedback, condom-catheter and pharmacological treatment) or more invasive approaches (implantation of sub-urethral sling or artificial urinary sphincter and the injection of periurethral bulking agents) (6, 7).

The problem associated with the anti-incontinence devices, especially artificial sphincters, is that to produce urethral occlusive force to achieve urinary continence, chronically occluded urethral wall, undergoes structural weakening, contributing to occurrence of atrophy and/or erosion (3). The urethral atrophy occurs by the decrease in the diameter of the urethra, leading to the insidious return of urinary incontinence. Erosion results in the migration of a component of anti-incontinence device into urethral lumen (8, 9) which is clinically expressed by perineal discomfort, vaginal discharge, hematuria, dysuria and urinary infection (10).

There is a growing interest in the search for alternatives that could reduce erosion rates and urethral atrophy after the implantation of these devices.

The use of biomaterials around urethra to act as protective barriers have been stimulated and currently used in clinical practice. In this regard, bacterial cellulose (BC), a biomaterial, has demonstrated efficacy as a driver cell and inductor in healing process (11, 12). Studies have proven its safety and efficacy in experimental and clinical models (11-13).

The objective of this study was to validate the application of bacterial cellulose membrane as urethral strengthening wrap, in an animal model in order to substantiate its clinical application as a protecting barrier to the urethra following the implantation of anti-incontinence devices based in morphometric and histological aspects of BC membrane integration.

## MATERIALS AND METHODS

### Animal model and experimental design

Wistar rats (n=40), weighing from 205g to 320g (250.12±17.26g), were used in the present study. Animals were kept in an appropriate environment with temperature and humidity control, day-night cycle artificially established for periods of 12 hours with free access to drinking and feed ad libitum. This study followed the principles governing the Code of Experimental Ethics and Laws for Protection of Animals, according to the standards in Brazil, receiving full approval from the Ethics Committee on Animal Experimentation of the Center for Biological Sciences, UFPE according to the process No. 23076.020552/2012-06.

Animals (n=40) were divided into four groups, with 10 animals per group, and named as follows: Group 1 (G1)=Sham; Group 2 (G2)=Bacterial Cellulose; Group 3 (G3)=Silicon; Grupo-4 (G4)=Bacterial Cellulose + Silicon. Half of the animals in each group were sacrificed at 4 and 8 months in order to evaluate short and long term outcomes.

### Surgical Procedure

The animals were identified and weighed prior to the surgical procedure. The anesthetic procedure was performed according to the standard operating procedures of the Nucleus of Experimental Surgery.

Animals were attached to the operating Table in the supine position. The abdominal antisepsis was performed with chlorhexidine solution. An abdominal midline incision of ±3cm was done above the pubic symphysis. Access to the abdominal cavity occurred by sequential dissection of the anatomical planes. The bladder and urethra were then exposed. With the use of scissors, a small suburethral space (±5mm) was created, just below the bladder neck to give passage to silicon tape and/or BC membrane. The control animals had the same procedure without placement of any material.

The silicon tapes and BC membrane were standardized in 3mm width and 7mm in length. Thicknesses were respectively 0.05mm for silicon and 0.54mm for BC. The membrane was wrapped around the urethra, and left in place. There was no need to fix it with suture since it is auto-adhesive. Contrariwise, silicon tapes after wrapped around the urethra were anchored with 5-0 Vycril. The abdominal wall was closed in two planes with 4-0 chromic catgut. Antibiotics were not used.

In the postoperative period, the animals were kept in an animal facility, in the same conditions. Five rats per group were killed four months after surgery. The second half (20 rats) had the same procedure after eight months. The killing process was done by intra peritoneal injection of sodium thiopental followed by intracardiac lethal dose of barbiturate. After the longitudinal access to the abdominal cavity, the bladder and urethra were removed en bloc.

### Summary of bacterial cellulose membrane

The BC membrane used in our study is a polysaccharide obtained by bacterial synthesis from sugarcane molasses in a process developed at the Experimental sugarcane Station of the Federal Rural University of Pernambuco (UFRPE), involved in the study. This material was supplied packed in isopropyl alcohol and then sterilized with gamma rays at the Metrology Laboratory of the Department of Nuclear Energy (UFPE), following pre-established concepts of surgical sterilization. Silicon membranes were donated sterile and in the pre-established dimensions by Medicone© Innovation for Health Ltd. (Cachoeirinha, RS, Brazil).

### Histological Analysis

The study material was sent to the Pathology Department which conducted the process of preparation of the slides. After fixed in formalin, cross section of the urethra was done just below the bladder neck. This segment was embedded in ethanol in increasing concentrations, diaphanized by xylene and impregnated with liquid paraffin. Fragments included for analysis were oriented in such a way that cross sections could be obtained perpendicular to the major axis of the urethra. The slides were stained with Hematoxylin-Eosin (HE) and Massom’s trichrome.

The evaluation of the slides and the capture of the images was done with the bright field microscope and immunofluorescence Axio Imager. M2m/Zeiss, connected to the digital camera AxioCam HRc/Zeiss, responsible for transferring images to a computer. The capture of the images was done through the ZEN-2012/Zeiss software.

### Measuring the intensity of the inflammatory reaction

All the measurements were performed by a blinded observer and the slides were randomly selected. Microscopic evaluation aimed to register the presence of neutrophils, lymphoplasmocytes, multinucleated giant cells (MNGC) and granulomatous infiltrate from the HE staining, in a semi-quantitative analysis. The intensity of the inflammatory reaction was graded as absent, mild, moderate and severe. This graduation was based on the following criteria [15]: 0-absent, with less than 5% of the examined area; 1-Lightweight, reaction involving between 5-25% of the examined area; 2-moderate, compromising between 25-70%; 3-Severe reaction, involving more than 70% of the area analyzed.

### Measuring Urethral Wall Height

**Figure 1:**
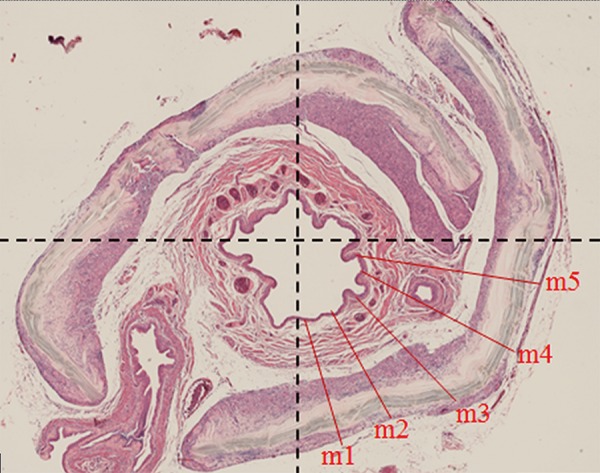
The urethral wall was measured in the lamina propria to the outer limit of the muscular layer according with the image. The area was divided in four quadrants to obtain the average of 20 measurements for each animal, making up 5 measurements in each of them.

To perform the measurements of the urethral wall, the area was divided in four quadrants to obtain the average of 20 measurements for each animal, making up 5 measurements in each of them (Figure-1). The images were captured with an increase of 5x in slides stained by HE. The measurements were made through the program Image J45 (National Institute of Health, Bethesda, MD, USA). For the different groups, the measurement of the thickness of the urethral wall was made starting from the lamina propria from the area in close contact with the urothelium, until the last outer muscle layer.

### Density of Blood Vessels

The density of blood vessels was made according to a method previously described for the quantification of microvessel density and the development of in vivo implants (14). Thus, the bladder neck wall cuts images were stained with HE and captured at 400x magnification and loaded into the image J software version 1.45. A contiguous area of 10.000µm^2^ implant was then drawn using the image J and all the vessels in the region bounded, in its light containing red blood cells, were counted.

The density of blood vessels was determined by dividing the number of vessels by the implant area and the results expressed as number of vessels/mm^2^. In samples from normal regions of the urethral wall, the vessel density was assessed in the lamina propria, defined as the loose connective tissue between the urothelial basal membrane and the inner portion of the muscle layer.

### Deposition of Collagen

Collagen intensity analysis was semi-quantitative and based on the same criteria described for analysis of the inflammatory response. The Masson method for staining provided an analysis of the concentration of collagen fibers present in the implant area and periurethral tissue.

### Statistical analysis

Statistical analysis was done by using Graph Pad Prism 5.0® software (Graph Pad Software Inc. USA). The values of the above study parameters were statistically evaluated for verification and confirmation of conditions such as adhesiveness and integration of the cellulosic membrane urethral wall, compared with silicon tape.

Parametric continuous variables (height of urothelium and urethral wall) were compared using the t test. For non-parametric (density of blood vessels) the Mann-Whitney test was applied. Scores (adhesiveness and integration, collagen deposition and answers inflammatory) were compared using the chi-square Pearson test. Statistical significance was set at p≤0.05. The statistical tests were performed using the GraphPad Prism 5.0 Program® (GraphPad Software Inc., USA).

## RESULTS

After the killing process, it was observed during the dissections that there was greater adherence to adjacent tissues including epiploic migration in G3. The animals in G1 showed the lowest adherence. Removed parts were stored in 10% buffered formalin before being sent to the pathology department.

The urethral epithelium responded similarly to the presence of both materials when applied alone, at the 4-months analysis. In the group of eight months, there was a reduction of epithelium height in G2 (30±1μm) and increase in G3 (51±2μm) when compared to G1 group (45±1μm). However, in G4 it was observed reduction in epithelium height when compared to the group followed for 4 months (24±1) and 8 months (33±3) (Table-1).

**Table 1:** Urethral Wall and Urothelium Height.

Wall Height (mm)	Sham		Bacterial Cellulose		Silicone		BC + Sil.	
Follow-up	4 montds	8 montds	4 montds	8 montds	4 montds	8 montds	4 montds	8 montds
Urethral	0.40±0.07	0.51±0.15	0.51±0.08	0.53±0.10 ^b^	0.58±0.12	0.41±0.10	0.50±0.14	0.37±0.11 ^d,f^
Urothelium	0.041±0.003	0.045±0.001	0.041±0.003	0.030±0.001 ^a,b^	0.034±0.002	0.051±0.002	0.024±0.001^d,e,f^	0.033±0.003 ^d,e,f^

Values expressed as Mean±SD. Student's t test significant if p≤0.05, to ^a^BC≠Sham; ^b^BC≠Silicone; ^c^Silicone≠Sham; ^d^BC≠BC+Sil.; ^e^Silicone≠BC+Sil; ^f^BC+Sil.≠Sham.

**NA**=Not applicable, **BC**=Bacterial Cellulose; **Sil.**=Silicone. Urethral wall p-value ^b^=0.0249; ^d^=0.0020; ^f^=0.0414.

Urothelium height p-value at 4 months: ^b^=0.058; ^d^=0.0009; ^e^=0.0103; ^f^=0.0037 and at 8 months: ^a^=0.0001; ^b^<0.0001; ^d^=0.3446; ^e^=0.0020; ^f^=0.0108.

Vasculogenesis in the implant with BC was similar between 4 and 8 months (4.44±0.57µm^2^ and 4.93±1.32, respectively), with guidance from the peripheral region toward the central region (centripetal) of the remaining material when compared to the group receiving the silicon (4 months: 0.53±0.34µm^2^, 8 months: 1.60±0.55µm^2^) (Table-2).

**Table 2:** Density of Blood Vessels in the implant area.

Density (µm^2^)	Sham		Bacterial Cellulose		Silicone		BC + Sil.			

Follow-up	4 months	8 months	4 months	8 months	4 months	8 months	4 months		8 months	

							BC	Sil.	BC	Sil.
Vasculogenesis	1.90±0.36	2.27±0.43	4.44±0.57^a,b^	4.93±0.13^b^	0.53±0.34	1.60±0.55^c^	5.76±0.12^f^	0.68±0.37 ^d,e,f^	2.48±0.10^f^	1.04±0.43 ^d,e,f^

Values expressed as Mean±SD. Mann Whitney test, significant if p≤0.05, to ^a^BC≠Sham; ^b^BC≠Silicone; ^c^Silicone≠Sham; ^d^BC≠BC+Sil.; ^e^Silicone≠BC+Sil; ^f^BC+Sil.≠Sham.

**NA**=Not applicable. BC: Bacterial Cellulose; **Sil.:** Silicone. Vasculogenesis p-value at 4 months: ^a^0.0159; ^b^0.0357; ^f^0.0159 for BC; ^d^0.0079; ^e^0.0357; ^f^0.0032 for Sil. and at 8 months: ^b^0.0571; ^c^0.0286; ^f^0.0159 for BC and ^d^0.0357; ^e^0.0159; ^f^0.0317 for Sil.

**Figure 2:**
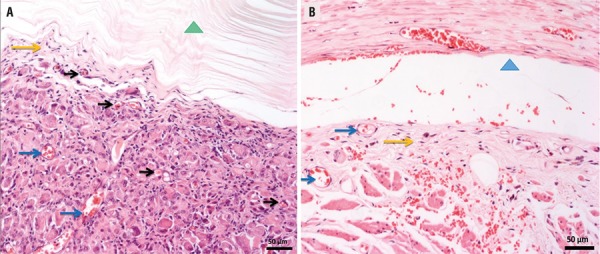
Photomicrography of the implant-tissue interface. A) Bacterial Cellulose group and B) Silicone group after 8 months. (∆) BC) (∆) Area where the silicon implant had occupied; (→) Blood Vessels; (→) vascular congestion; (→) inflammatory cells. Staining with hematoxylin and eosin. Note: Silicone implants did not resist the histological processing, for that reason the area appears as an empty space.

Inflammatory infiltrates became more strongly present from the periphery towards the central portion of the implant in the BC group (80% of the samples with moderate inflammation, at 4 months), with lymph nodes, without having, however, the confluence of the same. At 8 months the inflammatory response was mild (100% of the samples graduated as 1) (Supplementary Table-1, Figure-2).

**Supplementary Table 1:** The intensity of the inflammatory reaction.

Scores (%)	Sham		BC		Sil		BC + Sil.			

Follow-up (months)	4	8	4	8	4	8	4 months		8 months	

							BC	Sil.	BC	Sil.
**0**	100	100	0	0	67	100	0	25	0	100
**1**	0	0	20	100	33	0	25	0	20	0
**2**	0	0	80	0	0	0	75	0	80	0
**3**	0	0	0	0	0	0	0	0	0	0
p value			a0.0044 b0.0154	a0.0143 b0.0455			f0.0076	f<0.0001	f0.0078	f<0.0001

Values expressed as percentage (%). Chi-square test, significant if p≤0.05, to ^a^BC≠Sham; ^b^BC≠Silicone; ^c^Silicone≠Sham; ^d^BC≠BC+Sil.; ^e^Silicone≠BC+Sil; ^f^BC+Sil.≠Sham. **NA**=Not applicable. **BC**= Bacterial Cellulose; **Sil.**= Silicone.

In the tissue insertion in the urethra/silicon (G3), inflammatory infiltrates were dispersed (33%) and absent in 67% of samples at 4 months and absent all samples at 8 months (Supplementary Table-1, Figure-2).

**Figure 3:**
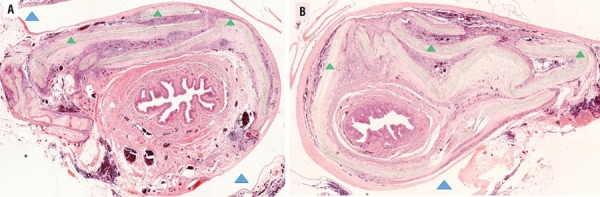
Rats urethra photomicrograph cross section showing bacterial cellulose plus silicon implant after 4 months (A) and after 8 months (B). (∆) Area where the silicon implant had occupied; (∆) bacterial cellulose implants. Staining with hematoxylin and eosin (10X). Note: Silicone implants did not resist the histological processing, for that reason the area appears as an empty space

In animals receiving BC remaining material was observed in the central area of the implant and in the more peripheral regions, especially periurethral, with moderate formation of CGMN (at 4 months) and intense (after 8 months). Small and medium vessels were also observed in this area. In the groups that received silicon, it was observed a fibrous capsule with the presence of CGMN only at 4 months (Figure-3).

In G4 group, it was observed intense CGMN formation between these materials. Histologically, it was seen increased collagen deposition (thin mature collagen fibers) in the group with BC when compared to the group receiving silicon (Supplementary Table-2).

**Supplementary Table 2:** Deposition of the Collagen in Periurethral area.

Scores (%)	Sham		BC		Sil		BC + Sil.			

Follow-up (months)	4	8	4	8	4	8	4 months		8 months	

							BC	Sil.	BC	Sil.
**0**	NA	NA	0	0	0	0	0	0	100	0
**1**			100	0	0	0	100	0	0	0
**2**			0	100	0	0	0	0	0	0
**3**			0	0	100	100	0	100	0	100
**p** *value*			^**a**^0.0044 ^**b**^0,0154	^**a**^0.0143 ^**b**^0,0455			^**f**^0.0076	^**f**^<0.0001	^**f**^0.0078	^**f**^<0.0001

Values expressed as percentage (%). Chi-square test, significant if p≤0.05, to ^a^BC≠Sham; ^b^BC≠Silicone; ^c^Silicone≠Sham; ^d^BC≠BC+Sil.; ^e^Silicone≠BC+Sil; ^f^BC+Sil.≠Sham. **NA**=Not applicable. **BC:** Bacterial Cellulose; **Sil:** Silicone.

**Figure 4:**
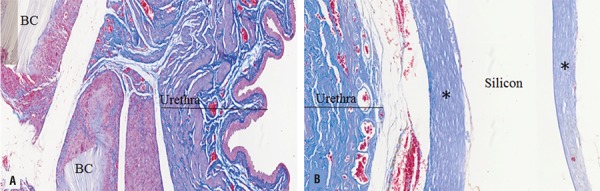
Photomicrography of collagen deposition in bacterial cellulose implants (A) and silicone implants (B), both after 8 months. Masson’s Trichrome Staining Protocol for Collagen Fibers (20X). *Thick collagen fibers. Note: Silicone implants did not resist the histological processing, for that reason the area appears as an empty space

There was intense presence of fibroblasts, although not quantified, especially at the region adjacent to the remaining BC membrane and light close to the fibrous capsule involving the silicon implant (Figure-4).

## DISCUSSION

Biomaterial is any natural or synthetic substance with the capacity to integrate into the tissue receiver, given a particular therapeutic purpose. Research community is engaged in the search for the ideal model. Among many requirements, it should be non-carcinogenic, easy acquisition and low cost and not associated with immunogenic reaction, qualifying as safe structure to several clinical applications (15). Collagen is the most common used matrix in medical practice, specially because it has proven adequate biocompatibility (16, 17).

In the present study, we used a cellulosic matrix, that has already been evaluated in biomechanical testing and biocompatibility as well as in cytotoxicity assays as a urethral reinforcing wrap in rats by evaluating its integration and remodeling to the host tissue (13, 18, 19). One of the main questions that we consider of interest in this evaluation is the change in the urethral wall layers thickness. We found that at 4 months, statistically similar behavior concerning urethral thickness when comparing BC (G2) and silicon (G3). This trend changes at 8 months, when there has been an increase of this measurement in the G2 when compared to the silicon group (p=0.0249). The results in G1 are supported in the literature in an article in which the author, employing porcine retail of small intestinal submucosa (SIS) in urethral fistula repair induced in rabbits, produced similar results, however without detailing the steps in this process and its effects on the urethral layers (20). The urethral wall, when measured from the lamina propria to the outer limit of the muscular layer, showed very different pattern. The later results (8 months) showed that there is significant structural gain in the isolated BC group when compared to the other groups that received the implant.

In G4 implant group when compared to the Sham group, it was found significant reduction in the layers thickness. This was the group where this phenomenon was more evident. On the other hand, the inflammatory reaction has declined significantly changing from moderate to light, in both opportunities of observation. In the BC group, the presence of CGMN and residual cellulosic material could be seen at eight months. In addition, at this time, presence of blood vessels of small and medium-sized implant in the region was evident. The G3 group showed lower inflammatory reaction than the G2, but a fibrous capsule formation was found at 4 months. The double implant group shows intense CGMN infiltration between silicon and urethra. It is well known that silicon is reactive, inducing foreign body reaction and encapsulation. However, this behavior has been seen when the material is implanted in host tissue intimacy, such as in slings (20-22).

Vasculogenesis was significantly present in the BC group when compared to groups where the silicon was used in both phases of the study. This was also observed in G1. At the same time, the presence of collagen at the implant area with the BC, ranged from mild to moderate, with the presence of mature collagen fibers, while identifying infiltrated unquantified fibroblasts around the residual cellulose. This was different from that observed in the groups, which received silicon alone, where there was intense collagen deposition, at different periods of measurement. It is well known that the biocompatibility of a material is validated by the degree of inflammatory reaction of vascularized tissue at the implant area (23).

This answer can range from inconsistent with the presence of fibrotic capsule formed and no new vessels, to consistent, with integration of mature collagen, vasculogenesis and inflammatory reaction that does not compromise the integration of the material to the host (20, 21).

The process of biocompatibility is induced through the recruitment of inflammatory cells in the implant area, which in turn contributes to the onset of neovascularization and consequent nutritional intake, necessary for the survival and transformation of the implanted matrix (23-25). It is worth to emphasize that in the inflammatory process itself cellularity is gradually replaced by fibroblasts and, therefore, collagen deposition, serves as a platform basis for the appearance of a new tissue structure (24).

In our experiment, BC induced the appearance of fibroblasts at the implant area, which was in equilibrium with collagen deposition, without tendency to encapsulation. This phenomenon was observed with the use of silicon. The literature on this subject is scarce. However, clinical study results suggest that silicon in direct contact with the urethral tissue increases the risk of erosion, regardless of the pressure applied on the area (25).

In other studies with the BC, it has been found intense inflammatory response in the early phase of the incorporating process, which decreases with time, as more collagen is incorporated without evidence of encapsulation. This phenomenon is interpreted by the authors as a sign that endorses the condition of biocompatibility of the material (11, 12).

Analyzing the different phases of BC incorporation to the host tissue our final impression is that the level of collagen deposition parameters, vasculogenesis and structural increase in urethral wall thickness lead to the belief that this may represent new perspective for longer survival of artificial implants in urology and other areas. Since the BC membrane is a natural product obtained from renewable source with low cost, may be considered as a future new material to be used in urology area.

## CONCLUSIONS

The BC was well integrated into the tissue receptor, contributing to its architecture remodeling and strengthening based on the morphometric and histological aspects of BC membrane integration. We can therefore accept that this new material may be a future option to prevent urethral damage.
